# Comparative and molecular analysis of MRSA isolates from infection sites and carrier colonization sites

**DOI:** 10.1186/s12941-018-0260-2

**Published:** 2018-03-15

**Authors:** Khaled R. Alkharsah, Suriya Rehman, Fatimah Alkhamis, Amani Alnimr, Asim Diab, Amein K. Al-Ali

**Affiliations:** 1 Department of Epidemic Diseases Research, Institute for Research and Medical Consultations (IRMC), Imam Abdulrahman Bin Faisal University (IAU), P.O. Box 1982, Dammam, 31441 Saudi Arabia; 2 Department of Microbiology, College of Medicine, Imam Abdulrahman Bin Faisal University (IAU), P.O. Box 1982, Dammam, 31441 Saudi Arabia; 3 Department of Biochemistry, College of Medicine, Imam Abdulrahman Bin Faisal University (IAU), P.O. Box 1982, Dammam, 31441 Saudi Arabia

**Keywords:** MRSA, SCCmec, MLST, Carrier, Infection, Saudi Arabia, CA-MRSA, HA-MRSA

## Abstract

**Background:**

Methicillin resistant *Staphylococcus aureus* (MRSA) constitutes a major global health concern causing hospital and community acquired infections. A wide diversity of MRSA genotypes are circulating in geographically related regions. Therefore understanding the molecular epidemiology of MRSA is fundamental to design control and clearance measures.

**Methods:**

A total of 106 MRSA isolates from infection (51) and carrier colonization sites (55) are characterized genetically based on SCCmec and MLST genotyping methods in addition to detection of PVL, TSST-1 and enterotoxins.

**Results:**

Sccmec-IV was the most frequently detected genotype (77.3%) followed by genotype V (13.2%) and III (9.4%). SCCmec-IVa was more prevalent among the carrier group (p value 0.002). CC80 was the most commonly identified clonal complex (CC). CC6 and CC22 were significantly more prevalent among the carrier group (p value 0.02 and 0.01, respectively). PVL was highly prevalent among the isolates (58.5%). PVL was detected in 70.6% of isolates from infection sites and 47.3% of isolates from carriers. All strains were sensitive to vancomycin, however, MRSA strains isolated from infection sites had significantly higher MICs compared to strains isolated from carrier colonization sites (p value 0.021). Five new sequence types mainly from the carrier group were identified and described in the study.

**Conclusions:**

MRSA population is genetically very diverse among carriers and infected individuals. With SCCmec type IV being most prevalent, this suggests a community origin of most MRSA strains. Therefore very well designed surveillance and clearance strategies should be prepared to prevent emergence and control spread of MRSA in the community.

## Background

Methicillin-resistant *Staphylococcus aureus* (MRSA) causes a wide variety of infections including life threatening infections. Consequently, MRSA is a global concern and it is one of the biggest threats in a health care facility [1]. The emergence of *S. aureus* strains resistant to methicillin and other categories of β-lactam antimicrobials was reported as early as 1960s and was later attributed to the expression of a protein that binds penicillin with low affinity (PBP2a) [2]. PBP2a is encoded by *mecA* gene (a small 2007 bp), a mobile extrinsic genetic element that is carried on a genomic island called staphylococcal cassette chromosome mec (SCCmec) [3]. According to the International Working Group on the Classification of Staphylococcal Cassette Chromosome Elements (IWG-SCC), eleven genotypes of MRSA have been identified based on the type of SCCmec [4]. SCCmec types I-III are generally detected in hospital acquired MRSA (HA-MRSA), which are the strains reported to cause hospital associated infections and outbreaks [5]. Other SCCmec types (IV, V or VII) are more frequently detected in community acquired MRSA (CA-MRSA), which cause infections in otherwise healthy individuals who had not been previously hospitalized [5]. However, CA-MRSA strains are increasingly reported in hospitals causing health-care associated infections [6]. Data from clinical and epidemiologic studies provide convincing indication that attribute the increased virulence of CA-MRSA over the HA-MRSA to the *lukF*-*PV* and *lukS*-*PV* (PVL) genes, which make the Panton–Valentine leukocidin (PVL) [7]. PVL causes cell lysis and is considered as a genetic symbol for CA-MRSA strains [8], however, PVL positive HA-MRSA has been reported in different studies [7].

MRSA strains could be also grouped into biologically meaningful groups called clonal complexes (CC) based on sequencing fragments from seven housekeeping genes in a method called multi locus sequence typing (MLST) [9]. CC showed geographically distinct distribution. For example, sequence types (ST) such as ST5 and ST8, belonging to CC5 and CC8 respectively, are commonly found in the United States and Japan, whereas strains belonging to the clonal complexes CC22 are more commonly found in Europe and Australia [10]. The ST80 (CC80) strain has been detected widely in the Middle East [11].

Hospitalization appears to present a patient with a major risk of infection with *S. aureus*. However, studies have shown that about one-fifth to one-third of the population carry *S. aureus* in their nose and about 2–3% of people carry MRSA [12]. In health care workers, this percentage increases to approximately 5% [13]. *S. aureus* carriers are another major risk factor and source of infection. The transmission of MRSA in hospitals and community needs to be better understood to determine the prevalence and peculiarity of circulating MRSA. Studies conducted in European hospitals on MRSA carriers have shown large variations in genetic diversity and in the prevalence of MRSA strains [14, 15]. Screening and identification of hospitalized MRSA carriers will result in a more effective control of MRSA.

In the current study, we have characterized MRSA strains isolated from carriers or infected individuals from the Eastern Province of Saudi Arabia and compared their genotypes and antibiotic susceptibility patterns. A better understanding of the circulating MRSA strains will result in a greater control and management of the disease.

## Methods

### Study design and bacterial isolates

A cross-sectional study was conducted from January 2010 to September 2011 at King Fahd hospital of the University (KFHU) in Al-Khobar in the Eastern Province of Saudi Arabia. A total of 106 MRSA isolates were collected from the microbiology laboratory of the hospital during the period of the study. All of these isolates were identified using standard microbiology procedures were confirmed using Vitek II (biomerieux, France). The isolates were confirmed for their resistance to oxacillin and cefoxitin using the disc diffusion method as described below. Clinical and demographic data were collected from patients’ medical records.

Ethical approval for the study was obtained from the Institutional Review Board at the University of Dammam (IRB-2013-08-023).

### Antimicrobial susceptibility testing

Twenty-four antibiotics were used in disc diffusion method on Mueller–Hinton agar (Oxoid, Hampshire, England) according to the Clinical Laboratory Standard Institute [16]. All antibiotic discs were purchased from Oxoid (Oxoid, Hampshire, England). Vancomycin susceptibility testing was performed by the E test strips (AB Biodisk, Sweden) [16].

### Molecular genotyping

#### Extraction of DNA

Bacterial DNA was extracted using Qiagen DNA extraction kit (Qiagen, Hilden, Germany) according to the manufacturer’s instructions. Extracted DNA was stored at − 80 °C till the time of analysis.

#### Detection of mecA and pvl genes

A multiplex PCR previously developed by Al-Talib et al. [17] was used to identify methicillin-resistant *Staphylococcus* by detecting the *mecA* gene. Additionally this multiplex PCR detects the presence of *lukS* gene of the Panton–Valentine leukocidin. To identify the genus *Staphylococcus* and to discriminate *S. aureus* from coagulase negative staphylococci, the primer-cocktail comprised primers targeting the 16S rRNA gene and the *femA* gene, respectively [17]. The internal control primers, originally included in Al-Talib protocol, were not included in our master mix. A laboratory isolate previously known to be positive for *mecA* and *PVL* genes was used as positive control. A water negative control was used with each run.

#### Detection of other staphylococcal virulence toxins

Two sets of primers were used in two multiplex PCR reactions (A and B) for detection of the staphylococcal enterotoxins genes A to E, toxic shock syndrome toxin 1 (tsst-1), and exfoliative toxins A and B according to Mehrotra et al. [18].

#### SCCmec typing

We followed a multiplex PCR protocol developed by Ghaznavi-Rad et al. for SCCmec genotyping [19]. The method uses nine pairs of primers to detect the SCCmec genotypes (I–III, IVa–IVd, IVh, and V) in addition to primers for *S. aureus* identification and detection of methicillin resistance *mecA* gene [19]. The PCR program was followed as described by Ghaznavi-Rad et al. [19]. The PCR product was run on 1.8% metaphor agarose (Lonza, Rockland, USA) at 25 V overnight in 0.5 × TBE buffer for good separation and size-based discrimination of the DNA bands.

#### MLST genotyping and sequence analysis

The multi locus sequence typing (MLST) protocol for *S. aureus* was followed according to the instructions on the MLST.net website (http://saureus.beta.mlst.net/). The allelic profile of each isolate was obtained and compared to the database to obtain the sequence type (ST). eBurst algorithm (http://saureus.beta.mlst.net/#eBURST) was used to categorize MLST into groups and clonal complexes (CC).

#### Data analysis

Statistical analysis was performed using SPSS version 23 and OpenEpi software. Phylogenetic tree of the STs was performed by eBurst.

## Results

The 106 MRSA isolates included in this study were obtained from 39 females (36.8%) and 67 males (63.2%) with an average age of 27.1 years (Table 1). Fifty-one strains (48.1%) were isolated from infection sites, including wound (16%), abscess (14.2%), skin and tissue infections (5.7%), and lower respiratory tract infections (4.7%). MRSA strains isolated from ear, eye and throat infections were less frequent (1.9% each). One strain was isolated from cerebrospinal fluid (0.94%) and one from a patient with a urinary tract infection (0.94%). Fifty-five strains (51.9%) were isolated from colonization sites such as nose (40.6%), throat (6.6%), axilla (2.8%) and groin (1.9%). MRSA strains isolated from colonization sites were isolated from Patients hospitalized for diseases not related to MRSA infection. The detection of MRSA in these patients was performed as a part of infection control policy which placed patients suspected or confirmed to be MRSA positive under contact isolation until decolonization is proven after successful prophylactic nasal mupirocin ointment and chlorhexidine body wash therapy. There was no statistically significant correlation between MRSA isolation from infection or colonization sites on the basis of age, duration of hospitalization, ICU admission, or outcome of treatment (Table 1).

**Table 1:** Frequency of MRSA isolates from carrier colonization sites and infection sites with demographic and clinical data

	Carrier (n = 55)	Infection (n = 51)	p value	OR (95% CI)
Gender				
Male	32	35	0.27	0.64 (0.28–1.42)
Female	23	16		
Age (years)^a^				
≤ 30	34	32	0.9	0.96 (0.43–2.12)
> 30	20	18		
ICU admission^b^				
Yes	51	48	0.67	0.53 (0.02–7.21)
No	2	1		
Duration of hospitalization (days)				
≤ 7	24	20	0.65	1.12 (0.55–2.63)
> 7	31	31		
Treatment outcome^c^				
Discharged rescreened negative	26	27	0.36	0.58 (0.17–1.84)
Discharged rescreened positive	10	6		

^a^One sample from each group has no data

^b^Two samples from each group have no data

^c^37 patients discharged without further screen

All MRSA isolates were positive for *mecA*, *femA*, and *sa442* genes. The most frequently detected SCCmec genotype was SCCmec-IV (77.3%) followed by SCCmec-V (13.2%), and III (9.4%). Further subtyping of SCCmec-IV revealed that subtypes IVa and IVc are the only subtypes prevalent among the isolates (41.5 and 58.5%, respectively) (Table 2). SCCmec-IVa genotype was more frequently found in MRSA isolated from the carriers compared to infected individuals (p value 0.002, OR 3.84) (Table 2).

**Table 2:** Molecular characterization of MRSA isolates from infection sites and carrier colonization sites

	Genotype	Carrier (55)	Infection (51)	p value	OR (95% CI)
SCCmec	III	3	7	0.17	0.37 (0.07–1.48)
	IVa	25	9	0.002	3.84 (1.59–9.81)
	IVc	21	27	0.13	0.55 (0.25–1.20)
	V	6	8	0.49	0.66 (0.20–2.1)
MLST^a^	CC1 (ST1, ST772)	5	8	0.30	0.53 (0.15–1.76)
	CC5 (ST5, ST149)	4	5	0.64	0.71 (0.16–2.96)
	CC6 (ST6)	6	0	0.02	Undefined
	CC8 (ST8,ST239, ST241)	3	8	0.091	0.31 (0.06–1.19)
	CC22 (ST22)	10	1	0.01	10.7 (1.70–242)
	CC30 (ST30)	0	2	0.23	Undefined
	CC45 (ST46)	2	0	0.27	Undefined
	CC80 (ST80,ST1440)^b^	21	23	0.43	0.73 (0.33–1.59)
	CC88 (ST88)	3	2	0.76	1.38 (0.20–12)
	CC97 (ST97)	1	1	0.95	0.91 (0.02–36.08)
PVL	Pos	26	36	0.02	0.38 (0.17–0.84)
	Neg	29	15		
*sea*	Pos	16	22	0.14	0.54 (0.24–1.22)
	Neg	39	29		
*seb*	Pos	7	1	0.04	7.18 (1.06–168.6)
	Neg	48	50		
*sec*	Pos	0	0	NA	NA
	Neg	55	51		
*sed*	Pos	3	0	NA	NA
	Neg	52	51		
*see*	Pos	11	6	0.26	1.86 (0.63–5.88)
	Neg	44	45		
*eta*	Pos	0	0	NA	NA
	Neg	55	51		
*etb*	Pos	0	0	NA	NA
	Neg	55	53		
*tst*	Pos	8	7	0.91	1.07 (0.35–3.34)
	Neg	47	44		
Average vancomycin MIC		0.81	1.26	0.021^$^	(0.35–0.55)

*sea, seb, sec, sed, and see* staphylococcal enterotoxin A, B, C, D, and E

*eta and etb* exfoliative toxin A and B

*tst* toxic shock toxin

^a^One of the isolates in infection group was a singleton in eBurst and could not be allocated to any CC

^b^Includes also the 4 new STs from 6 samples according to eBurst

^$^p value (two-tailed) from Hartley’s f test for equality of variance

**Fig. 1:**
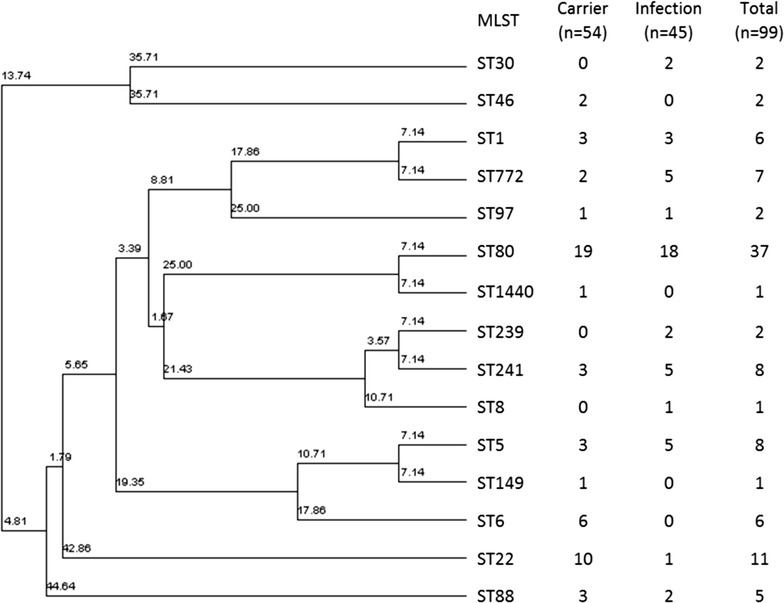
Phylogenetic analysis of the MRSA MLST-genotypes and their frequency among carrier and infection groups. 7 new types are not included in the analysis

MLST genotyping of the MRSA isolates showed that ST80 is the most frequently encountered genotype in our study (34.9%) followed by ST22 (10.4%) (Table 2 and Fig. 1). ST6 and ST22 were more frequently detected in the carrier group than the infection group (p value = 0.02 and 0.01, respectively) (Table 2). Other MLST genotypes, such as ST30, ST1930, ST239 and ST241 were more frequently detected among the infected patients but were statistically not significant (Table 2).

Toxic shock syndrome toxin 1 (TSST-1) was present in 14.2% of the isolates. Staphylococcal enterotoxin A (SEA) was the most frequently detected enterotoxin (35.8%) (Table 2). SEB was significantly more prevalent among the carrier group compared to the infection group (p value 0.04) (Table 2).

Panton-Valentine leucocidin (PVL) was positive in 58.5% (62/106) of the total MRSA isolates. It was more frequently detected in the infection group of MRSA isolates (70.6%) compared to the carrier group isolates (47.3%) (p value 0.02, Table 2). However, when analyzing the prevalence of PVL among the MRSA isolates from infection sites only based on whether these isolates are CA or HA-MRSA, PVL was more frequently detected in the CA-MRSA isolates but this difference was not statistically significant (Table 3).

**Table 3:** Prevalence of PVL among the MRSA isolates from infection sites

	CA-MRSA^a^ (n = 32)	HA-MRSA^a^ (n = 19)	p value	OR (95% CI)
PVL				
Pos	24	12	0.4	1.7 (0.49–6.1)
Neg	8	7		

^a^According to our hospital infection control policy, the CDC classification of MRSA was used to classify MRSA as CA or HA

Clonal complex 80 (CC80) was the most frequent clonal complex detected in the study (41.51%) (Table 4) followed by CC1 (12.26%), CC8 and CC22 (10.38% each), CC5 (8.49%), CC6 (5.66%), CC88 (4.71%). CC30, CC45, and CC97 were the least frequent isolates in the study (1.89% each) (Table 4). One new isolate was not possible to classify into any of the clonal complexes (0.94%) and seem to constitute singleton. Most of the clonal complexes harbored SCCmec type IV (IVa or IVc). SCCmec in ST239 and ST241 (CC8) was of type III only (Table 4).

**Table 4:** Affiliation of MRSA isolates MLST, SCCmec, PVL, and TST to clonal complexes

CC	MLST	SCCmec	PVL	TST	Carrier	Infection	Total
CC1	ST1	IVa	Neg	Neg	3	2	5
		IVc	Pos	Neg	0	1	1
	ST772	IVa	Pos	Neg	0	1	1
		V	Pos	Neg	2	4	6
CC5	ST5	IVc	Neg	Pos	0	2	2
			Pos	Neg	1	1	2
		V	Neg	Neg	2	0	2
			Pos	Neg	0	2	2
	ST149	IVa	Neg	Neg	1	0	1
CC6	ST6	IVa	Neg	Neg	6	0	6
CC8	ST8	IVa	Pos	Neg	0	1	1
	ST239	III	Neg	Neg	0	2	2
	ST241	III	Neg	Neg	3	5	8
CC22	ST22	IVa	Neg	Pos	7	1	8
			Pos	Neg	1	0	1
		IVc	Pos	Neg	2	0	2
CC30	ST30	IVa	Pos	Pos	0	1	1
		IVc	Pos	Neg	0	1	1
CC45	ST46	IVc	Neg	Neg	2	0	2
CC80	ST80	IVa	Neg	Neg	3	0	3
			Pos	Neg	1	0	1
		IVc	Neg	Neg	1	0	1
			Pos	Pos	0	2	2
			Pos	Neg	13	15	28
		V	Pos	Neg	1	1	2
	ST1440	IVc	Pos	Neg	1	0	1
CC88	ST88	IVa	Neg	Neg	0	1	1
			Pos	Pos	1	0	1
			Pos	Neg	2	1	3
CC97	ST97	V	Neg	Neg	1	1	2

All MRSA isolates were sensitive to vancomycin, however, MRSA strains isolated from infection sites had significantly higher minimum inhibitory concentrations (MIC) compared to strains isolated from carrier colonization sites (p value 0.021) (Table 2). There was no other significant difference in the antibiotic susceptibility pattern between MRSA isolates from infection sites and carrier colonization sites (Table 5). PVL was not associated with any antibiotic susceptibility pattern.

**Table 5:** Frequency of antibiotic resistance among the isolated strains

Antibiotic	Carrier (n = 55)	Infection (n = 51)	p value	Odds ratio (CI 95%)
Amikacin	7 (12.7%)	10 (19.6%)	0.35	0.60 (0.20–1.74)
Azithromycin	26 (47.3%)	27 (52.9%)	0.57	0.80 (0.37–1.72)
Cefoxitin	55 (100%)	51 (100%)	NA	NA
Chloramphenicol	53 (96.4%)	49 (96.1%)	0.94	1.08 (0.11–10.71)
Ciprofloxacin	27 (49.1%)	31 (60.8%)	0.24	0.62 (0.29–1.36)
Clindamycin	3 (5.5%)	5 (0.1%)	0.43	0.53 (0.10–2.44)
Doxycycline	21 (38.2%)	19 (37.3%)	0.92	1.04 (0.47–2.3)
Erythromycin	43 (78.2%)	40 (78.4%)	0.98	0.99 (0.38–2.52)
Fosfomycin	3 (5.5%)	1 (0.02%)	0.41	2.86 (0.29–77.36)
Fusidic acid	55 (100%)	51 (100%)	NA	NA
Gentamycin	5 (9.1%)	10 (19.6%)	0.13	0.41 (0.12–1.30)
Kanamycin	38 (69.1%)	41 (80.4%)	0.19	0.55 (0.22–1.34)
Levofloxacin	8 (14.5%)	10 (19.6%)	0.50	0.70 (0.24–1.97)
Linezolid	0 (0%)	0 (0%)	NA	NA
Ofloxcacin	7 (12.7%)	9 (17.6%)	0.50	0.68 (0.22–2.03)
Oxacillin	55 (100%)	51 (100%)	NA	NA
Vancomycin	0 (0%)	0 (0%)	NA	NA
Quinu_Dalfo	9 (16.4%)	5 (0.1%)	0.34	1.79 (0.56–6.31)
Rifampicin	2 (3.6%)	1 (0.02%)	0.66	1.88 (0.14–56.7)
Sulphamethoxazol	3 (5.5%)	2 (0.04%)	0.74	1.41 (0.20–12.27)
Teicoplanin	53 (96.4%)	50 (98%)	0.66	0.53 (0.02–7.19)
Tetracycline	36 (65.5%)	36 (70.6%)	0.58	0.79 (0.34–1.81)
Tigecycline	53 (96.4%)	46 (90.2%)	0.23	2.85 (0.54–22.1)
Tobramycin	4 (7.3%)	7 (13.7%)	0.30	0.50 (0.12–1.83)

Five new MLST alleles from seven samples were identified in this study (Table 6). The isolates were obtained from scalp abscess, endotracheal aspirate, breast abscess, skin swab (cellulitis), urine, wound swab and one sample from nasal swab (Table 6). These alleles produced new sequence types (STs) that have not been previously described in the MLST database. All except one were PVL positive and harbored the SCCmec-IV (Table 6).

**Table 6:** Characteristics of the new ST alleles found in the study

MLST genes							CC	SSCmec	PVL	Type of sample
*arcC*	*aroE*	*glpF*	*gmk*	*pta*	*tpi*	*yqil*				
1	3	1	14	4	51	10	80	IVc	Pos	Scalp abscess
1	3	1	14	New	51	10	80	IVc	Pos	Endotracheal aspirate
1	New	1	14	11	51	10	80	IVc	Pos	Nasal swab
1	New	1	14	11	51	10	80	IVc	Pos	Breast abscess
1	New	1	14	11	51	10	80	IVc	Pos	Skin swab (cellulitis)
3	New	1	1	4	4	3	ND	IVa	Neg	Urine
1	3	1	14	11	New	10	80	IVc	Pos	Wound swab

*ND* not determined, *Pos* positive, *Neg* negative

## Discussion

Extensive knowledge of the circulating MRSA strains is an important prerequisite for control and surveillance measures. Little is known about the genetic diversity of MRSA in the Middle East and the Gulf area. The Arabian Gulf countries attract a wide range of working manpower from different countries, which in turn enriches the microbial diversity in these countries. In the current study we characterized the MRSA isolates from the Eastern Province in Saudi Arabia and compared the molecular types of these isolates from infection sites and from carrier colonization sites.

We did not find difference between the two groups regarding duration of hospitalization. Other studies have reported that MRSA infection prolongs the duration of hospitalization [20]. However, the type of infections in our study comprises mainly skin infections (abscess and wound) which do not necessarily require hospitalization. Interestingly, we found some molecular types confined to either the carrier or infection sites.

The most frequently detected SCCmec was type IV (IVa and IVc), which is in line with a previous study from the same region [21]. Type IV SCCmec is typically detected in MRSA isolates from samples outside hospitals [4, 5]. In our study SCCmec type IV strains were equally detected in carriers and from infection sites (Table 2). SCCmec subtype IVa was significantly associated with strains isolated from carrier colonization sites. Sccmec type V, which is commonly associated with CA-MRSA infections [5], was equally detected in isolates from carriers and infection sites in our study. Similarly the sequence types ST6 and ST22 (except for one case) were only detected in MRSA isolates from carriers in our study.

PVL was highly prevalent among our isolates (56.5%), an observation which has been reported elsewhere [21, 22]. However, other studies reported a lower prevalence (8–12%) [23, 24]. Moreover, PVL was significantly associated with MRSA strains isolated from infection sites compared to carriers. Among MRSA isolates from infection sites, PVL was more frequently found in CA-MRSA, which is consistent with the literature [25]. Nonetheless, our study found that a large number of HA-MRSA isolates from infection sites harbor PVL (63.1%). This indicates that PVL-positive strains are invading hospitals and causing infections, which is in line with previous reports [26]. Despite the controversial role of PVL as virulence factor in MRSA pathogenesis [27, 28], it seems to play an important role in the successful evolutionary fitness of these strains [15]. Therefore, special control and clearance measure should be directed toward PVL-positive MRSA. Four MRSA strains were found to be simultaneously positive for both PVL and TSST-1 genes in our study, which is otherwise uncommon [29]. One of the four isolates was obtained from a patient with severe necrotizing pneumonia that required ventilation and developed sepsis and one isolate was obtained from deep cutaneous abscess. The other two isolates came from surgical wound infection and colonization sites. A similar finding has also been reported in the Middle East and other countries [29–32].

The most commonly identified clonal complex in our study was the CC80-MRSA-IV. This has been previously reported in several countries in the Middle East and North Africa and has also been reported from Riyadh in the central region of Saudi Arabia [11, 21, 22, 33–37]. This could indicate that it is the most common MRSA strain circulating in Saudi Arabia. This clone represents the European CA-MRSA clone. The spread of this clone in the Middle East and North Africa and the presence of sporadic cases in Europe suggest that this clone was introduced to Europe through migration from both the Middle East and North Africa. CC80-MRSA-IV isolates are usually PVL positive. However, PVL negative clones have been sporadically reported [11, 21, 35, 38, 39]. Four of the CC80-MRSA-IV isolates in our study were PVL negative. Since it has been suggested that PVL provides fitness to this isolate, in addition to the presence of this isolate in small numbers, it could be assumed that they are sporadic deletion variants. ST1440-IV was detected in only one sample in the present study. This isolate is very rare and has only been reported in Tunisia [40]. ST80-MRSA-V was identified in two cases in our study. This genotype has not been reported previously and seems to be a new isolate as all reported ST80 strains harbor the SCCmec genotype IV.

Two sequence types (ST1 and ST772) belonging to CC1 were identified in our study. Most ST1 strains were PVL negative and harbored SCCmec-IV, which may resemble the West Australian strain WA MRSA-1. The rest were PVL-positive strain, which may resemble the USA400 strain. ST772-MRSA-V is usually more distinct from the other CC1 isolates and is known as the Bengal Bay clone. This strain is more prevalent in India [41] and was also detected in many European countries, where it was linked to transmission associated with migration to these countries [42]. Similarly, the detection of this strain in Saudi Arabia could be explained by the presence of the large number of Indian workers in the country. The detection of ST772-MRSA-IV has not been previously reported and it was isolated from one case in our study.

The UK-MRSA-15 strain, which is also called Barnim Epidemic strain (C22-MRSA-IV PVL-negative), was detected in 7.5% of our isolates and was found more frequently among carriers. Similar to other reports, it differs from the UK-MRSA-15 strain in that it is positive for the *tst1* gene and has the SSC-IVa [22]. The other PVL-positive strain was detected in three cases and was confined to the carrier group. This strain has been reported in many other countries, including the neighboring United Arab Emirates [15].

Contrary to other studies from Riyadh in Saudi Arabia [21, 22], ST241-MRSA-III PVL-negative strain was the most frequently detected isolate belonging to CC8, which indicates interregional variation in the distribution of these strains. This isolate has been reported in Kuwait, Tunisia, Taiwan and Poland [43–46]. The other CC8 strains were the ST239-MRSA-III and the USA300 (ST8-MRSA-IV PVL-positive) were previously reported from Riyadh region [21, 22].

Similar to previous reports from Saudi Arabia, PVL-negative and positive CC5 strains harboring the SCCmec IV or V were identified in our study [21, 22]. These strains were isolated also in Qatar, Tunisia and Egypt [24, 44, 47, 48]. One sample in our study has genotypes similar to the Maltese strain (CC 5/ST149) [49].

All the CC6 isolates were detected in the carrier group and they were also reported previously in Riyadh and the United Arab Emirate [15, 22], which indicates that it is an established strain in the region.

Other clonal complexes such as CC30, CC45 and CC97 were isolated from 1.9% of the cases each. ST30-MRSA-IV PVL-positive resembles the US1100 (southwest pacific clone) and is widespread in Europe and the Gulf region [15, 50]. Interestingly, CC45-MSSA strains were previously detected in Saudi Arabia from nasal colonization carriers [51]. Therefore, it is possible that this strain has acquired the mecA gene and transformed to MRSA. CC45-MRSA-IV was also reported previously in one case from Riyadh in Saudi Arabia [21] and one case from Hong Kong. Similarly, the CC97-MRSA-V was sporadically reported from many countries, including the Middle Eastern region [15].

The phenomenon of vancomycin MIC creep was described by Wang et al. and confirmed by several reports worldwide [52]. In MRSA strains, high vancomycin MICs, which still lay within the susceptible range (≥ 1.0), was linked with poor clinical outcome and higher mortality [53]. In our cohort, isolates originating from infected sites, but not colonization sites, exhibited high MICs to vancomycin raising a concern of clinical efficacy of the drug as monotherapy in this case. The method used to measure the MIC, E test, may have contributed to the increased MICs values [54]. However, a recent meta-analysis by van Hal et al. has shown that despite the methodology applied, these MICs close to the break point of 2.0, such as the isolates representing infected sites in this study, carry poor prognosis irrespective of the infection site [55]. Appropriate alternatives in such circumstances include daptomycin in cases of bacteremia and Linezolid in pneumonias.

Five new sequence types from seven samples are reported in this study (Table 6). All except one were isolated from infection sites. They belong to CC80 according to the eBust website. Due to the diversity of nationalities working and living in the Gulf region, further studies should be conducted on MRSA to reveal and monitor the emergence of new strains.

## Conclusions

A highly diverse population of MRSA isolates is reported from both carriers and patients’ infection sites in our study from the Eastern Province in Saudi Arabia. Some of the isolate types were more prevalent among the carriers more than the infection sites. Therefore, screening and control measures should consider the polyclonal nature of the problem. A good understanding of the genetic spectrum of the MRSA isolates and its prevalence in the population calls for continuous surveillance and clearance measures. With SCCmec type IV being most prevalent; this suggests a community origin of most MRSA strains which is also supported by low resistance rates to clindamycin and sulfonamides. Therefore very well designed surveillance and intervention strategies, in particular restricting antimicrobial use, should be implemented to prevent emergence and control spread of MRSA in the community.

## Abbreviations

MRSA: methicillin resistant *Staphylococcus aureus*
*S. aureus*: *Staphylococcus aureus*
PBP2a: penicillin binding protein 2a
mecA gene: methicillin resistance gene
SCCmec: staphylococcal cassette chromosome mec
HA-MRSA: hospital acquired MRSA
CA-MRSA: community acquired MRSA
lukF-PV: leukocidin gene subunit F
lukS-PV: leukocidin gene subunit S
PVL: Panton–Valentine leukocidin
CC: clonal complexes
MLST: multi locus sequence typing
ST: sequence types
femA: factor essential for mecA
PCR: polymerase chain reaction
ICU: intensive care unit
MIC: minimum inhibitory concentration
